# Demographics, stage distribution, and relative roles of surgery and radiotherapy on survival of persons with primary prostate sarcomas

**DOI:** 10.1002/cam4.1872

**Published:** 2018-11-19

**Authors:** Jonathan D. Tward, Matthew M. Poppe, Ying J. Hitchcock, Brock O’Neil, Daniel J. Albertson, Dennis C. Shrieve

**Affiliations:** ^1^ Department of Radiation Oncology University of Utah School of Medicine Salt Lake City Utah; ^2^ Department of Urology University of Utah School of Medicine Salt Lake City Utah; ^3^ Department of Pathology University of Utah School of Medicine Salt Lake City Utah

**Keywords:** prostate sarcoma

## Abstract

**Background:**

Primary prostate sarcomas (PPS) are rare. Outcomes for this cancer have not been well characterized.

**Materials and Methods:**

Subjects with a PPS diagnosed between 1973 and 2014 were identified in the SEER database. Subjects were stratified by disease stage and types of therapies received. Disease‐specific survival (DSS) and Overall survival (OS) was estimated by Kaplan‐Meier analysis and cohorts were compared with a univariate and multivariable Cox regression.

**Results:**

The incidence of PPS among all prostate cancer diagnoses was 0.02%. Subjects younger than age 26 years at diagnosis represented 29% of cases, and 32% of primary prostate sarcomas were rhabdomyosarcoma histology.

**Rhabdomyosarcoma Histologies:**

The median age at diagnosis was 9 years. Between age 0‐25 years rhabdomyosarcoma accounted for 96.4% of primary prostate sarcoma diagnoses, after age 25 rhabdomyosarcoma represented 15% of new diagnoses. The 10‐year DSS and OS for rhabdomyosarcoma was 47% and 44%.

**Non‐Rhabdomyosarcoma Histologies:**

The median age at diagnosis was 71 years. The most common diagnoses were leiomyosarcoma (33%) and carcinosarcoma (28%). Localized, regional, or distant disease occurred in 40%, 34%, and 26% of cases. The 10‐year DSS and OS were 26% and 14%. In locally advanced cases, RT added to surgery trended toward improved DSS (*P* = 0.10).

**Conclusions:**

Disease‐specific survival and OS for non‐rhabdomyosarcoma histologies appear inferior to those of rhabdomyosarcoma. The addition of RT to surgical resection may improve DSS in locally advanced non‐rhabdomyosarcoma. This is the largest report of the incidence, stage distribution, and survival for this extremely rare urologic malignancy providing valuable prognostic information.

## 1 INTRODUCTION

Prostate sarcomas are extremely rare and have been estimated to account for fewer than 0.1% of primary prostate cancers.^1^ Prostate sarcomas originate from the mesenchymal tissues which include the fibromuscular stroma, smooth muscle, blood vessels, paraganglia, and nerves.^2^ Prior reports attempting to characterize the various histologies and outcomes of men with primary prostate sarcomas have been limited to institutional case series and case reports.^2^, ^3^, ^4^, ^5^, ^6^, ^7^, ^8^, ^9^, ^10^, ^11^, ^12^, ^13^, ^14^, ^15^, ^16^, ^17^, ^18^, ^19^, ^20^, ^21^, ^22^, ^23^, ^24^, ^25^, ^26^, ^27^, ^28^ Rhabdomyosarcoma is the most common diagnosis in juveniles, but reportedly rare in adults.^2^ The largest case series previously reported that includes non‐rhabdomyosarcoma prostate sarcomas is restricted to adults and only includes 38 subjects.^4^ The purpose of this study is to evaluate the demographics, stage distribution, and outcomes for both children and adults diagnosed with primary prostatic sarcomas.

## 2 PATIENTS AND METHODS

Access to The National Cancer Institute's (NCI) Surveillance, Epidemiology, and End Results (SEER) Program database was granted to the authors after receipt of a signed data use agreement. The University IRB has determined that data in this dataset does not rise to the level of “human subjects research,” under the federal Common Rule, 45 CFR Part 46 and University policies, and, therefore formal IRB review was waived. Subjects were identified in the National Cancer Institute's Surveillance, Epidemiology, and End Results Program Database using the SEER‐Stat program. Subjects with an International Classification of Diseases for Oncology 3rd edition (ICD‐O3) topology code of 69.1 (prostate) and histology codes of 8800‐9059 were selected from records obtained between the years 1973‐2013. The Intergroup Rhabdomyosarcoma Pretreatment Clinical Staging System (IRS) was calculated for cases with adequate information. Cases were further stratified based upon histology into Rhabdomyosarcoma and non‐rhabdomyosarcoma and by primary treatment type (surgery, radiation, combined surgery, and radiation). Survival estimates were performed using the Kaplan and Meier method.^29^ Comparisons between treatment groups were made with Cox regression analysis.^30^

## 3 RESULTS

There were 1 165 297 persons diagnosed with prostate cancer of any histology between the years 1973 and 2014. Of those, 295 persons were diagnosed with primary prostate sarcoma, for an incidence of only 0.02% of all prostate primaries. There were 141 245 persons diagnosed with sarcoma at any body site during this same era, but only 0.21% originated in the prostate. The median and average follow‐up time of persons who did not die of sarcoma was 68 and 90 months, respectively. Demographics of the study population can be found in Table 1. The median, 2, 5, and 10 years overall and disease‐specific survival (DSS) estimates by disease extent are listed in Table 2. The tumor size was reported in 77 cases (26.1%). Among these cases, the median tumor size was 70 mm (range 6‐180 mm). The PSA was recorded in 17 cases (5.8%) and the median PSA value at diagnosis was 4.2 ng/mL (range 0.5‐40.7 ng/mL).

**Table 1:** Demographics of the study population

	Non‐Rhabdo	Rhabdo
	N = 200	N = 95
Median age	71 y	9 y
Age		
>25 y old	197 (98.5%)	14 (14.74%)
<25 y old	3 (1.5%)	81 (85.26%)
Race		
White	170 (85%)	79 (83.2%)
Other	17 (8.5%)	8 (8.4%)
African American	12 (6.0%)	8 (8.4%)
Unknown	1 (0.5%)	0 (0.0%)
Definitive therapy by extent^a^		
Localized	47 (23.5%)	11 (11.6%)
Neither surgery or radiation	4 (8.5%)	(0%)
RT alone	(0%)	6 (54.5%)
Surgery alone	33 (70.2%)	(0%)
Surgery + Radiation	9 (19.1%)	4 (36.4%)
Unknown	1 (2.1%)	1 (9.1%)
Regional	40 (20%)	15 (15.8%)
Neither surgery or radiation	1 (2.5%)	(0%)
RT alone	2 (5%)	8 (53.3%)
Surgery alone	16 (40%)	3 (20%)
Surgery + Radiation	18 (45%)	4 (26.7%)
Unknown	3 (7.5%)	(0%)
Distant	30 (15%)	32 (33.7%)
Neither surgery or radiation	5 (16.7%)	4 (12.5%)
RT alone	7 (23.3%)	20 (62.5%)
Surgery alone	11 (36.7%)	2 (6.3%)
Surgery + Radiation	5 (16.7%)	5 (15.6%)
Unknown	2 (6.7%)	1 (3.1%)
Unknown extent	83 (41.5%)	37 (38.9%)
Neither surgery or radiation	11 (13.3%)	8 (21.6%)
RT alone	6 (7.2%)	10 (27%)
Surgery alone	49 (59%)	6 (16.2%)
Surgery + Radiation	15 (18.1%)	10 (27%)
Unknown	2 (2.4%)	3 (8.1%)
Grade		
Well differentiated	10 (5%)	2 (2.1%)
Moderately differentiated	25 (12.5%)	0 (0%)
Poorly differentiated	42 (21%)	6 (6.3%)
Undifferentiated/anaplastic	45 (22.5%)	10 (10.5%)
Unknown	78 (39%)	77 (81.1%)
Cancer sequence number		
One primary only	119 (59.5%)	90 (94.7%)
1st of 2 or more primaries	28 (14%)	4 (4.2%)
2nd of 2 or more primaries	47 (23.5%)	1 (1.1%)
3rd of 3 or more primaries	5 (2.5%)	(0%)
4th of 4 or more primaries	1 (0.5%)	(0%)

a Extent prior to 1998 categorized as unknown.

**Table 2:** Overall (OS) and disease‐specific (DSS) survival estimates for primary prostate sarcoma by stage presentation

	Non‐Rhabdo		Rhabdo	
	OS	DSS	OS	DSS
All stages	N = 200		N = 95	
2 y	48%	53%	59%	61%
5 y	26%	34%	46%	48%
10 y	14%	26%	44%	47%
Median survival	24 mo	28 mo	30 mo	36 mo
Localized	N = 47		N = 11	
2 y	66%	66%	89%	89%
5 y	44%	51%	76%	76%
10 y	23%	41%	76%	76%
Median survival	42 mo	111 mo	Not reached	Not reached
Regional	N = 40		N = 15	
2 y	50%	54%	85%	85%
5 y	27%	35%	68%	68%
10 y	10%	18%	68%	68%
Median survival	24 mo	28 mo	Not reached	Not reached
Distant	N = 30		N = 32	
2 y	35%	38%	52%	52%
5 y	16%	20%	26%	28%
10 y	8%	10%	22%	24%
Median survival	12 mo	13 mo	26 mo	26 mo

### 3.1 Rhabdomyosarcoma

**Figure 1:**
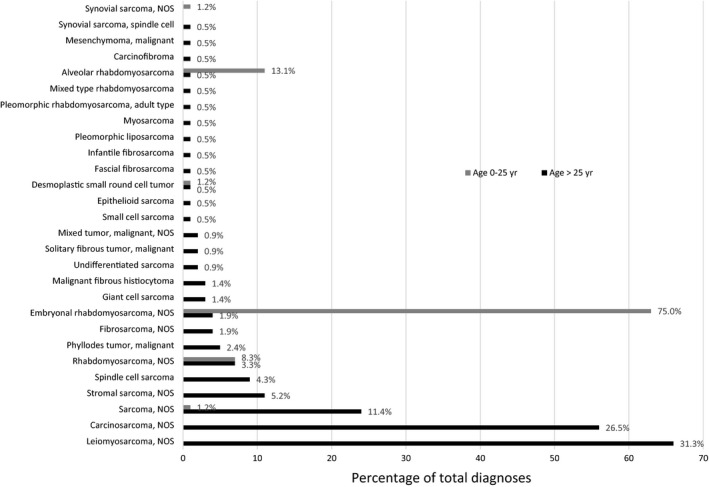
Distribution of prostate sarcoma histologies by age cohort

Rhabdomyosarcoma accounted for 32.2% of diagnoses overall. Of the rhabdomyosarcomas, 70.5% were embryonal and 12.6% were alveolar (Figure 1). Rhabdomyosarcoma accounted for 96.4% of prostate sarcoma diagnoses in subjects 25 years or younger but was only 6.6% of diagnoses in men older than age 25. The median age at diagnosis of a rhabdomyosarcoma patient was 9 years and the mean age was 15 years, with a range of 0‐87 years.

### 3.2 Extent of disease

Of the 58 subjects with discrete extent of disease information (only available for subjects diagnosed after 1998), 19% were localized only, 26% had locoregional disease, and 55% were metastatic at diagnosis. The IRS clinical stage could be calculated for 36 subjects. There were 2, 22, and 12 subjects with IRS stage 2, 3, and 4 disease, respectively. Fifteen subjects had either carcinomatosis or the site of metastasis discretely characterized. Carcinomatosis was present in nine subjects. There were six subjects with metastatic disease where the sites of metastases were discretely characterized: four had lung metastases (67%), two subjects (33%) had bone metastases, and no brain or liver metastases were reported. Because surgical margin status was not encoded, the Intergroup Rhabdomyosarcoma Clinical Grouping System and Children's Oncology Group Risk Group Stratification could not be calculated.

### 3.3 Survival

**Figure 2:**
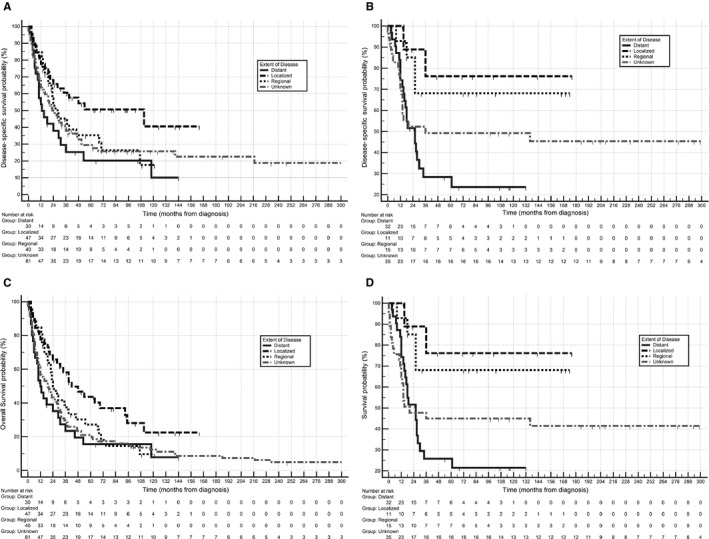
A, Disease‐specific survival, non‐rhabdo cases. B, Disease‐specific survival, rhabdo cases. C, Overall survival, non‐rhabdo cases. D, Overall survival, rhabdo cases

There were of 95 persons diagnosed with Rhabdomyosarcoma and 50 deaths were observed. Only four of the observed deaths were due to causes other than the cancer. Most disease‐specific deaths occurred within 5 years following therapy (Figure 2). For 30 subjects with local or locoregional disease only (including four subjects diagnosed prior to 1998, and 26 after 1998), 16 (53%) had radiotherapy, 4 (13%) had surgery, and 9 (30%) had surgery and radiotherapy combined. Those having radiation therapy versus surgery plus radiotherapy had virtually identical 10‐year DSS, 75%, and 78%, respectively. The four subjects who had surgery without radiation had no observed sarcoma deaths. The 5‐year overall survival (OS) and DSS of the 14 subjects diagnosed with rhabdomyosarcoma after age 25 was 32.7% and 35.4%, respectively.

### 3.4 Non‐Rhabdomyosarcoma

Non‐Rhabdomyosarcoma accounted for 67.8% of prostate sarcoma diagnoses overall (Figure 1). The median age at diagnosis was 71 years and the mean age was 68 years, with a range of 17‐97 years. The most common histology was leiomyosarcoma (33%), followed by carcinosarcoma (28%). No other histologic subtype exceeded 6% of diagnoses.

### 3.5 Secondary malignancy population

There were 41 (20.5%) non‐rhabdomyosarcoma persons in whom the prostate sarcoma was not their first primary malignancy. Among these men, 19 had a prior diagnosis of prostate adenocarcinoma, 11 of whom who had radiation therapy to the prostate. The remaining eight persons with a prior diagnosis of prostate adenocarcinoma were either diagnosed/treated with TURP or other non‐destructive prostate techniques. The second most common prior malignancy was bladder cancer (nine persons), only one of whom had been treated with radiation therapy. The remaining 13 prior malignancies included lung, colon, lymphoma, melanoma, peritoneal primary, rectum, and urethra. Of those 13, three individuals had been exposed to prior radiation (two rectal, one lung). Therefore, a total of 14 of the 41 (34%) subjects with a secondary prostate sarcoma were exposed to radiotherapy at a location (bladder, prostate, or rectum) that may have contributed to their prostate sarcoma development.

### 3.6 Extent of disease

Of the 117 subjects with extent of disease information, 40% were localized only, 34% had locoregional disease, and 26% were metastatic at diagnosis. There were nine subjects with metastatic disease where the sites of metastases were discretely characterized: four had lung (44%), two had liver (22%), one subject (11%) had bone metastases, and no brain metastases were recorded. The distribution of cases having disease confined to the prostate, seminal vesicle extension, bladder or rectum, or further extension is shown in Table S1.

### 3.7 Survival

**Figure 3:**
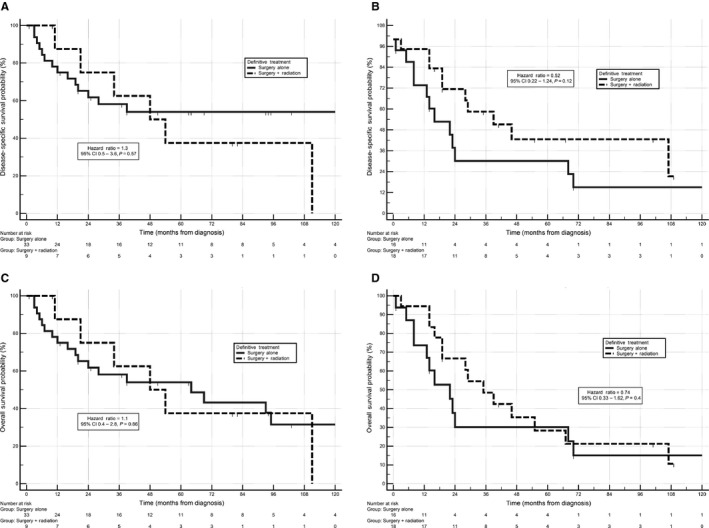
A, Disease‐specific survival, non‐rhabdo localized. B, Disease‐specific survival, non‐rhabdo regional. C, Overall survival, non‐rhabdo localized. D, Overall survival, non‐rhabdo regional

There were of 200 persons diagnosed with non‐rhabdomyosarcoma and 165 deaths were observed, with 125 deaths attributable to the cancer. For 88 subjects with local or locoregional disease, 2 (2%) had radiotherapy, 52 (59%) had surgery, and 25 (28%) had surgery and radiotherapy combined. Those having surgery plus radiation therapy versus surgery alone had 5‐year DSS of 55% and 42%, respectively. The DSS univariate hazard ratio for surgery plus radiation versus surgery alone for localized or locoregional disease combined was 0.90 (95% Confidence Interval 0.49‐1.66, *P* = 0.74). However, if one restricts the analysis to regional disease only, the hazard ratio for surgery plus radiation versus surgery alone trends toward a benefit for the addition of radiation (HR = 0.52, 95% Confidence interval 0.22‐1.23, *P* = 0.11; Figure 3). However, with only 18 and 16 subjects evaluable with regional disease receiving surgery or surgery plus radiation, there was not enough power to detect statistical significance.

### 3.8 Additional analyses in the adult population

Among subjects over age 25 with either localized or regional disease, there were 10 individuals with tumors <5 cm and 20 individuals with tumors ≥5 cm. There was a non‐statistically significant trend toward improved OS (5 year OS = 47% vs 32%, HR = 0.46, 95% CI 0.18‐1.22, *P* = 0.16) and DSS (5 year DSS = 47% vs 35%, HR = 0.49, 95% CI 0.18‐1.34, *P* = 0.21) for the smaller lesions.

A multivariable analysis in the persons over age 25 taking into account stage (localized, regional, or metastatic), tumor size, histologic group, type of definitive therapy, age at diagnosis, and year of diagnosis was performed (Table 3). Factors significantly associated with improved overall survival were localized disease or regional disease, younger age, and tumors classified as phyllodes or mixed tumors. These same factors were associated with improved DSS except for age and regional disease, although regional disease tended toward significance (*P* = 0.1). Definitive radiotherapy alone (as opposed to surgery, or surgery plus radiation) also had a trend toward inferior OS (HR = 1.88, 95% CI 0.95‐3.74, *P* = 0.072) and DSS (HR = 1.92, 95% CI 0.54‐1.83, *P* = 0.097), although this difference was not statistically significant.

**Table 3:** Multivariable analysis of potential prognostic factors on OS and DSS in the adult (age >25) population

	Overall survival		Disease‐specific survival	
	Hazard ratio (95% CI)	*P*‐value	Hazard Ratio (95% CI)	*P*‐value
Age	1.02 (1.01‐1.03)	**0.003**	1.01 (0.89‐4.16)	0.157
Definitive therapy				
Neither surgery nor radiation	Reference			
Radiation alone	1.88 (0.95‐3.74)	0.072	1.92 (0.54‐1.83)	0.097
Surgery alone	0.89 (0.52‐1.51)	0.668	0.99 (0.47‐1.89)	0.982
Surgery + Radiation	0.91 (0.5‐1.68)	0.768	0.94 (0.33‐2.82)	0.860
Unknown	1.1 (0.44‐2.73)	0.843	0.97 (0.2‐2.28)	0.951
Histology				
Rhabdomyosarcoma	Reference			
Fibroblastic and myofibroblastic tumors	0.75 (0.25‐2.21)	0.600	0.68 (0.17‐15.56)	0.529
Miscellaneous soft tissue sarcomas	2.28 (0.4‐12.88)	0.350	1.61 (0.06‐4.59)	0.681
Liposarcomas	0.4 (0.05‐3.4)	0.403	0.53 (0.58‐9.08)	0.561
Fibrohistiocytic tumors	2.12 (0.55‐8.19)	0.278	2.29 (0.28‐1.52)	0.238
Leiomyosarcomas	0.68 (0.31‐1.5)	0.341	0.65 (0.47‐2.5)	0.320
Unspecified soft tissue sarcomas	1.09 (0.5‐2.38)	0.837	1.08 (0.03‐0.82)	0.854
Phyllodes or mixed tumors	0.22 (0.06‐0.75)	**0.016**	0.17 (0.44‐2.39)	**0.027**
Other complex mixed and stromal neoplasms	1 (0.45‐2.21)	0.993	1.02 (0.62‐3.5)	0.961
Size				
<5 cm	Reference			
≥5 cm	1.63 (0.7‐3.8)	0.255	1.47 (0.59‐2.99)	0.378
Unknown size	1.53 (0.69‐3.38)	0.293	1.32 (0.18‐0.71)	0.500
Stage				
Metastatic	Reference			
Localized	0.38 (0.21‐0.7)	**0.002**	0.36 (0.31‐1.1)	**0.003**
Regional	0.56 (0.31‐1)	**0.050**	0.59 (0.48‐2.22)	0.098
Unknown	0.93 (0.46‐1.84)	0.826	1.03 (0.98‐1.05)	0.944
Year of diagnosis	1.01 (0.98‐1.03)	0.665	1.02 (0.98‐1.05)	0.334

Values in boldface represent *P*‐value ≤ 0.05.

There were 13 persons with rhabdomyosarcoma and 65 persons with leiomyosarcoma older than 25 years with local or locoregional disease. On univariable analysis, there was no significant difference for DSS (leiomyosarcoma vs rhabdomyosarcoma hazard ratio 0.67, 95% CI 0.26‐1.62, *P* = 0.30) or OS (HR = 0.83, 95% CI 0.39‐1.78, *P* = 0.61) observed. The multivariable analysis referenced above also showed no statistical difference for DSS (HR = 0.65, 96% CI 0.47‐2.5, *P* = 0.32) or OS (HR = 0.68, 95% CI 0.31‐1.5, *P* = 0.34) of leiomyosarcoma versus rhabdomyosarcomas in adults.

## 4 DISCUSSION

Due to its extreme rarity and histologic diversity, primary prostate sarcoma outcomes can only be practically studied with large population databases. Prior works have either restricted their case series to a single histology, like the more prevalent rhabdomyosarcoma^7^, ^8^, ^9^, ^10^, ^11^ or Leiomyosarcoma,^28^ to adult cases only,^4^, ^5^, ^6^, ^7^, ^8^, ^9^, ^10^, ^11^, ^17^, ^25^, ^28^, ^31^ or have not characterized the specific role of radiation in addition to surgery with regard to survival outcomes. The SEER database captures incidence and outcomes data on 28% of the United States population, allowing for a more detailed and well‐powered analysis of demographic information, stage distribution, and treatment outcomes than those of single institutional experiences. This study represents the largest case series assembled to date of persons diagnosed with primary prostate sarcomas, and one of the first to address the relative merit of radiation therapy in this population. Nevertheless, the SEER database does not track chemotherapy utilization, patterns of treatment failure, doses of radiation therapy used or treatment field designs, completeness of resection and surgical margin status, or molecular diagnostic information that may also be prognostic for sarcomas. Therefore, these important factors could not be included in our survival analyses.

The most common histologic type of prostate non‐rhabdomyosarcoma we identified was leiomyosarcoma. In our adult population, this represented 31% of diagnoses. This is consistent with the reports of case series from high volume centers like Memorial Sloan Kettering (MSK; 13/38 cases, 34%), MD Anderson (12/21 cases, 57%), and West China Hospital of Sichuan University (8/25 cases, 40%).^4^, ^5^, ^6^ The MSK group reported a statistically significant worse DSS for adult rhabdomyosarcoma over leiomyosarcoma in their series (HR = 3.00; 95% CI 1.13, 7.92; *P* = 0.027), which differs from our finding that DSS and OS for these histologies were statistically equivalent in our adult populations. Their study only had 12 and 13 individuals with rhabdomyosarcoma and leiomyosarcoma respectively, and the difference they observed might be due to imbalance in the stages, age, or other prognostic features in their respective cohorts which were unaccounted for in their analysis. Although we did adjust for age and stage and other covariates in our multivariable analysis, with only 13 subjects evaluable with adult rhabdomyosarcoma it was unlikely we would have statistical power to detect significant differences unless survival was substantially different. That being said, we did observe better survival among subjects with phyllodes or mixed tumors (seven subjects), which suggests an intrinsic biology that is less lethal than the other histologies. Nevertheless, with relatively small numbers of subjects in any histologic group, and multiple subgroup testing, any conclusion about variable survival among different histologies in the adult population should be viewed skeptically.

Our work confirms that virtually all the primary prostate sarcomas diagnosed in the pediatric and young adult population are rhabdomyosarcomas (RMS). RMS accounts for only 4.5% of pediatric malignancies (SEER Cancer statistics), with bladder and prostate often lumped together for analysis. Combined, bladder and prostate account for 5%‐11% of RMS cases.^32^, ^33^ RMS of the bladder and prostate are considered an unfavorable site and demonstrate a worse prognosis compared to other genitourinary sites.^33^ Minimal surgery is a prostatectomy; however a cystoprostatectomy or anterior pelvic exenteration is often required to achieve a gross resection. In the early studies performed by the Intergroup Rhabdomyosarcoma (IRS) cooperative group, surgery was the mainstay of local management, with radiation reserved for post‐operative residual disease or in cases to avoid a pelvic exenteration. The IRS‐I study (1972‐77) commonly included pelvic exenteration and reported a 3‐years DFS of 70%. The IRS‐II study (1978‐84) used neoadjuvant chemotherapy with the goal of reducing the need for pelvic exenteration and/or radiation therapy. The 3‐years DFS decreased to 55% in this trial yet bladder function did not improve compared to IRS‐1, 22% vs 23%.^34^, ^35^, ^36^ In the IRS‐3 study (1984‐91) radiation therapy was routinely used in all patients after week 6 of induction chemotherapy unless tumors could be completely resected without sacrificing the bladder. Patients retained their bladders in 60% of cases and had a 90% survival rate.^37^ In the IRS‐IV study (1993‐1997) this approach was continued (definitive chemotherapy and radiation) unless a complete surgery could be performed with bladder preservation. The intact bladder rate in this trial was 70%, however detailed bladder function questionnaires completed by patients revealed that only 40% of patients maintained normal bladder function.^38^, ^39^, ^40^ Sexual dysfunction has not been widely studied in boys having surgical management of prostate RMS, but is likely a common toxicity. Sexual and bladder dysfunction from prostate radiation are possible, but not well described. Radiation doses used for rhabdomyosarcoma are typically 50.4 Gy for gross disease compared that of non‐rhabdomyosarcoma (70 Gy) or prostate adenocarcinoma (>72 Gy). As such, it is possible that the lower doses required to control RMS in the prostate could lead to preservation of adequate sexual function, and is an important topic for future study.

Unfortunately, the SEER database does not release chemotherapy utilization data, and we therefore could not quantify its utilization and how it may have impacted our survival analyses. Furthermore, with fewer than 15 persons having either localized or regional rhabdomyosarcoma, statistical comparisons between surgery, radiation, or the combination for overall or disease‐specific cannot be performed due to the lack of statistical power.

Radiation therapy alone was seldom performed in persons with localized or regional non‐rhabdomyosarcoma disease. In the multivariable analysis, there was a suggestion that RT alone trended toward inferiority compared to surgical therapy in adults. However, there were fairly sizeable DSS curve separations for the addition of radiation to surgery in people with regional disease specifically. One wonders if the p value of 0.12 when comparing populations of only 16 and 18 persons would reach significance if the population size was larger. This may be consistent with the finding of De Bari et al^31^ who observed an improved 5 year local control (55% vs 31%, *P* = 0.02) and overall survival (59% vs 46%, *P* = 0.1) for persons receiving either neoadjuvant, adjuvant, or definitive radiotherapy relative to surgery alone in an adult population that included 61 patients treated in a network of 16 American and European Institutions. Radiation doses higher than those prescribed for soft tissue sarcomas (50‐60 Gy) are routinely delivered to the prostatic fossa for prostate adenocarcinoma (64‐72 Gy) with acceptably low risk of long‐term side effects. Nevertheless, combined chemo‐radiotherapy regimens for sarcomas designed to be radiosensitizing may offset the benefit of dose‐escalation. Since chemotherapy and radiation dose data are lacking from this database, we cannot evaluate the relative merits of dose‐escalation or radiosensitization at this disease site. Although radiation therapy may not have significant impact on DSS or OS in many cases, it may reduce local recurrence‐free survival, which has been proven in extremity sarcoma randomized trials.^41^, ^42^ We therefore recommend the administration of radiation after surgical resection for cases of locally advanced or regional prostate sarcoma.

## 5 CONCLUSIONS

Primary prostate sarcomas are a rare and heterogeneous group of histologies that present at varying stages of disease. DSS and OS for localized and regional rhabdomyosarcoma, have fairly good long‐term survival in the decade following diagnosis. Non‐rhabdomyosarcoma histologies, which are almost exclusively adult diagnoses, have poor long‐term DSS and OS, even when presenting with localized disease. Radiotherapy added to surgical removal of the prostate may improve DSS in adult patients, but radiotherapy alone may be inferior. This is the only study to date that can help prognosticate long‐term survival for subjects with prostate sarcoma by stage presentation and the relative contribution of radiation therapy relative to surgery. Given the poor outcomes and requirement for careful multidisciplinary planning in this niche malignancy, men afflicted with primary prostate sarcomas should ideally be managed at expert centers whenever practical.

## CONFLICTS OF INTEREST

None.
