# Supplementary material for: Demographics, stage distribution, and relative roles of surgery and radiotherapy on survival of persons with primary prostate sarcomas
Source: Cancer Med. 2018 Nov 19;7(12):6030–9. doi: 10.1002/cam4.1872 (PMC6308088; doi:10.1002/cam4.1872)
Supplement: Supplementary file 2 [file CAM4-7-6030-s002.docx]

|  | Localized | Regional | Distant | Unknown | ***Grand Total*** |
| --- | --- | --- | --- | --- | --- |
| **Turp or Biopsy only** | 9 | 4 | 5 |  | ***18*** |
| Negative Nodes | 8 | 3 | 4 |  | ***15*** |
| Positive Nodes |  | 1 | 1 |  | ***2*** |
| Unknown | 1 |  |  |  | ***1*** |
| **Confined to Prostate** | 14 | 3 | 3 | 2 | ***22*** |
| Negative Nodes | 14 | 2 | 3 | 2 | ***21*** |
| Positive Nodes |  | 1 |  |  | ***1*** |
| **Seminal Vesicle Invasion** | 1 | 2 |  | 1 | ***4*** |
| Negative Nodes | 1 | 2 |  | 1 | ***4*** |
| **Bladder or Rectal Invasion** |  | 11 | 3 |  | ***14*** |
| Negative Nodes |  | 10 | 2 |  | ***12*** |
| Positive Nodes |  | 1 | 1 |  | ***2*** |
| **Sidewall,colon, or bone** | 1 | 2 | 5 |  | ***8*** |
| Negative Nodes | 1 |  | 2 |  | ***3*** |
| Positive Nodes |  | 2 | 1 |  | ***3*** |
| Unknown |  |  | 2 |  | ***2*** |
| **Unknown** | 22 | 18 | 14 | 80 | ***134*** |
| Negative Nodes | 1 | 1 | 1 | 1 | ***4*** |
| Positive Nodes |  |  | 2 |  | ***2*** |
| Unknown | 21 | 17 | 11 | 79 | ***128*** |
| ***Grand Total*** | ***47*** | ***40*** | ***30*** | ***83*** | ***200*** |

Supplementary appendix table 1: Distribution of local extent and adenopathy by overall stage.
